# Oral administration of Ginkgolide B alleviates hypoxia-induced neuronal damage in rat hippocampus by inhibiting oxidative stress and apoptosis

**DOI:** 10.22038/ijbms.2018.26228.6569

**Published:** 2019-02

**Authors:** Wang Li, Shi Qinghai, Li Kai, Ma Xue, Niu Lili, Ran Jihua, Liu Zhengxiang, Li Xiaoling, Ge Di, Yang Qi, Deng Mengyun, Fu Jianfeng

**Affiliations:** 1 Clinical Laboratory Diagnostic Center, General Hospital of Xinjiang Military Region, Urumqi 830000, Xinjiang, China; 2 Clinical Laboratory Diagnostic Center, Changji State People’s Hospital, Changji, Xinjiang, China; 3 Clinical Laboratory Diagnostic Center, Sichuan Provincial People’s Hospital , Chengdu, Sichuan, China

**Keywords:** Apoptosis, Ginkgolide B, Hippocampus, Hypoxia, Oxidative stress injury

## Abstract

**Objective(s):**

The aim of this study is to explore the potential neuroprotective effects of Ginkgolide B (GB), a main terpene lactone and active component in *Ginkgo biloba*, in hypoxia-induced neuronal damage, and to further investigate its possible mechanisms.

**Materials and Methods:**

54 Sprague-Dawley rats were randomly divided into three groups: the untreated control group (n=18); the hypoxia group (n=18; exposed to 6000 m simulated plateau altitude for six days); and the GB group (n=18; intragastric administration of 12 mg/kg GB three days prior to rapid adaption to 6000 m and on the first two days of hypoxia). After hypoxia exposure for six days, we dissected out the brain hippocampi and performed hematoxylin and eosin staining, Nissl staining, and TUNEL staining. Homogenates of the hippocampi were used to test the oxidative stress indices including malondialdehyde (MDA), superoxide dismutase (SOD), glutathione (GSH), and catalase. Bax and caspase-3 expression in the hippocampal tissue was measured using qRT-PCR.

**Results:**

Treatment with GB before exposure to hypoxia could protect neural cells and increase the number of Nissl bodies. TUNEL and qRT-PCR results demonstrated that GB treatment could decrease apoptotic cells in different areas of the hippocampus. Antioxidant defense systems such as SOD, GSH, and catalase were decreased (*P*<0.05), and the concentration of MDA was reduced significantly in the hippocampi of rats of the GB group (*P*<0.05).

**Conclusion:**

GB could alleviate hypoxia-induced neuronal damage in rat hippocampus by inhibiting oxidative stress and apoptosis.

## Introduction

The oxygen-deficient environment of high-altitude areas such as plateaus are linked to an increased probability of developing “high-altitude symptoms”, and reduced physical performance capacity and cognitive abilities in mammals (1, 2). Hypoxia could decrease ATP synthesis and increase reactive oxygen species (ROS) (3). In addition, hypoxia could decrease the activity of the cellular antioxidant system, which could lead to oxidative stress (4). ROS promote the formation of toxic oxidation reaction products, which have a cytostatic effect due to cell membrane damage, and cause apoptotic or necrotic cell death. Various compounds, including superoxide dismutase (SOD), vitamins A, C, and E, catalase, glutathione (GSH), bilirubin, albumin, uric acid, and ceruloplasmin, play a role in maintaining the balance between ROS and antioxidants (5).

Hypoxia causes neuronal cell injury by oxidative stress (6-8). Inhibition of oxidative stress to prevent neuronal death could therefore be a novel therapeutic strategy. Ginkgolide B (GB), a major terpene lactone and active component of *Ginkgo biloba* (9), has neuroprotective effects in degenerative dementia and neurosensory disorders (10-12). It has been reported that *Ginkgo biloba *extract (13) could inhibit neuronal apoptosis under serum deprivation (14). Moreover, GB reduced ROS levels *in vivo*, suggesting that the raw extract contains antioxidants (15). Findings have also shown that GB treatment decreased ROS and intracellular calcium levels and activated Akt phosphorylation *in vivo* and *in vitro*, thereby decreasing cell viability and apoptosis (16). We previously found that GB could increase the cognitive ability of SD rats that were rapidly exposed to a simulated 6000 m plateau for six days (not published), based on the Morris water maze test. Thus, GB likely protects hippocampal neural cells under hypoxia. However, the molecular mechanisms underlying the neuroprotective effects of GB remain unclear. 

In this study, we aimed to examine the putative protective effect of GB against hypoxia-induced oxidative stress and apoptosis in the rat hippocampus. Our results could provide novel preventive therapeutic options for neuroprotection, particularly in individuals undergoing rapid initial adaptation to hypobaric hypoxia environments.

## Materials and Methods

***Establishment of animal models***

GB (CAS#15291-77-7, LOT: C31J6G2014, Purity > 98%) was purchased from Shanghai Yuanye CO, Ltd, China. Fifty-four specific-pathogen-free (SPF) Sprague-Dawley (SD) male rats (8-10 weeks old, weighing 220-250 g) were obtained from the Animal Experimental Center, Xinjiang Medical University (Urumchi, China). Rats were housed under humidity and temperature-controlled conditions and a 12 hr light/dark cycle with free access to food and water. All rats were randomly assigned to three experimental groups: group I (untreated control group, n=18); group II (hypoxia group, n=18; animals subjected to rapid ascent to 6000 m); group III (GB group, n=18; animals subjected to intragastric administration of 12 mg/kg GB three days prior to exposure to 6000 m simulated altitude). The care and use of animals for all experiments was in compliance with the Provisions and General Recommendation of the Chinese Experimental Animals Administration Legislation and was approved by the Science and Technology Department of Xinjiang Province. 

Group I rats fed in a standard environment. Group II and Group III rats were exposed to simulated hypobaric hypoxia for six days at 6,000 m in an animal decompression chamber (FLYDWC50-IA, Aviation Industry Corporation of China, China) where the temperature and humidity were maintained at 20 ± 2 ^°^C and 30 ± 5%, respectively. The rate of ascent to simulated high altitude was 40 m/sec, and pressure was maintained at 354 ± 2 mmHg. Fresh air was allowed to flow into the chamber at the rate of 5.5 l/min during the exposure. Group III rats were subjected to intragastric administration of GB (12 mg/kg) for three days before they were placed in the animal decompression chamber, and for the first two days after exposure to simulated hypobaric hypoxia. The dose of GB depended on previous experiments and the report of Yu Botao* et al. *(17).

**Figure 1:**
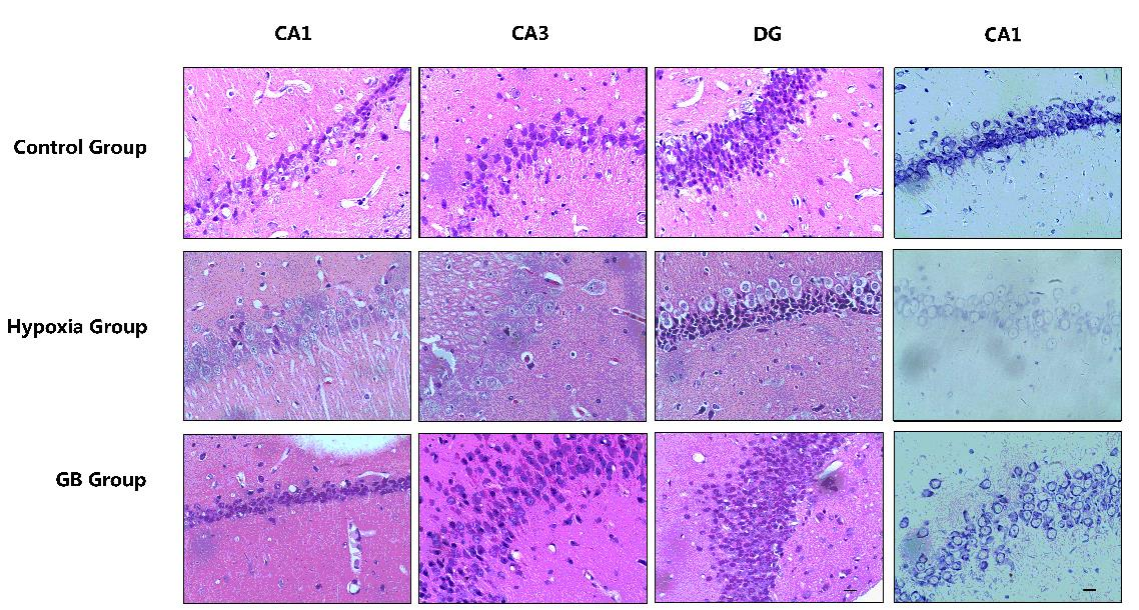
Morphological evidences prove that Ginkgolide B (GB) treatment could protect hippocampal neural cells

Representative 400× images of the HE-stained CA1, CA2, dentate gyrus (DG) areas of hippocampal sections and Nissl-stained CA1 areas of hippocampal sections from the three experimental groups. Scale bar: 100 μm

**Figure 2:**
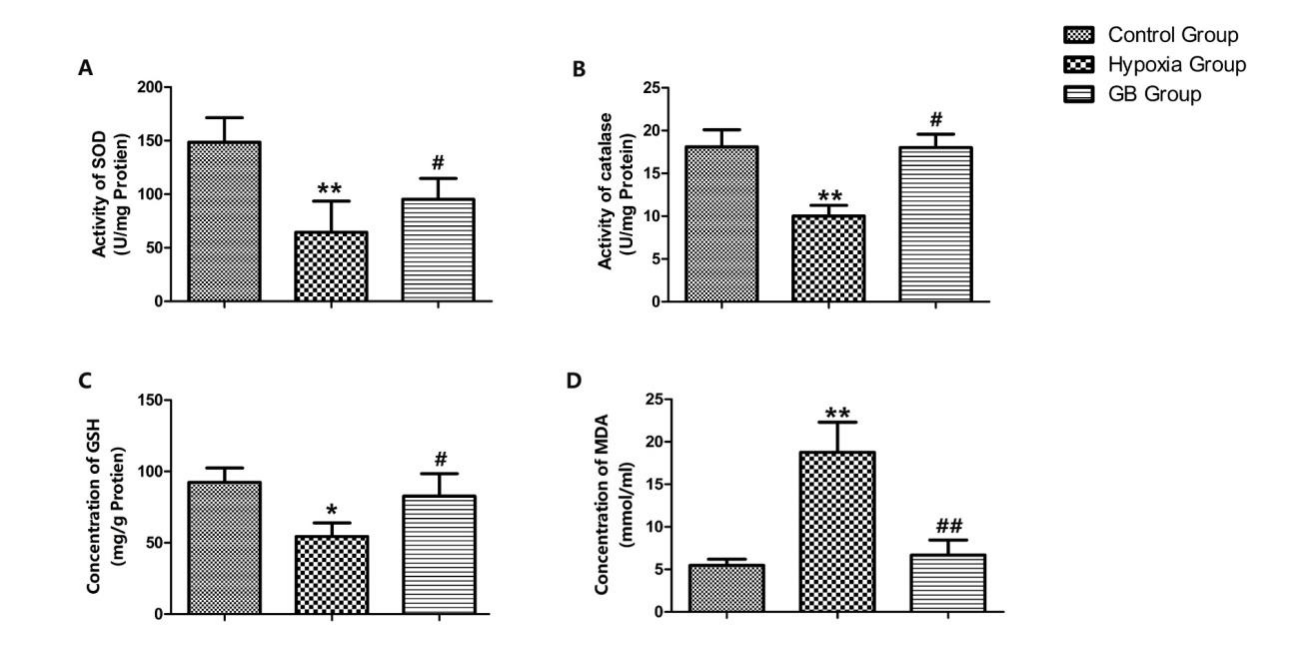
Ginkgolide B (GB) protects neurons by preventing apoptosis

(A) Representative 400× images of the terminal deoxynucleotidyl transferase-mediated dUTP end labeling (TUNEL)-stained hippocampal CA1 region showing fewer positive cells (marked by arrows) in the GB group compared to the hypoxia group. (B) Quantification of TUNEL-positive cells in the hippocampal CA1 region. The results are expressed as mean±SD. **P<0.01 vs. control group; ##*P*<0.01 vs. hypoxia group. (C) RT-PCR analysis of mRNA levels of Bax, Bcl-2, and caspase-3 (n=3 per group). *P<0.05, **P<0.01 vs. control group; #*P*<0.05, ##*P*<0.01 vs. hypoxia group, Scale bar: 100 μm

**Figure 3:**
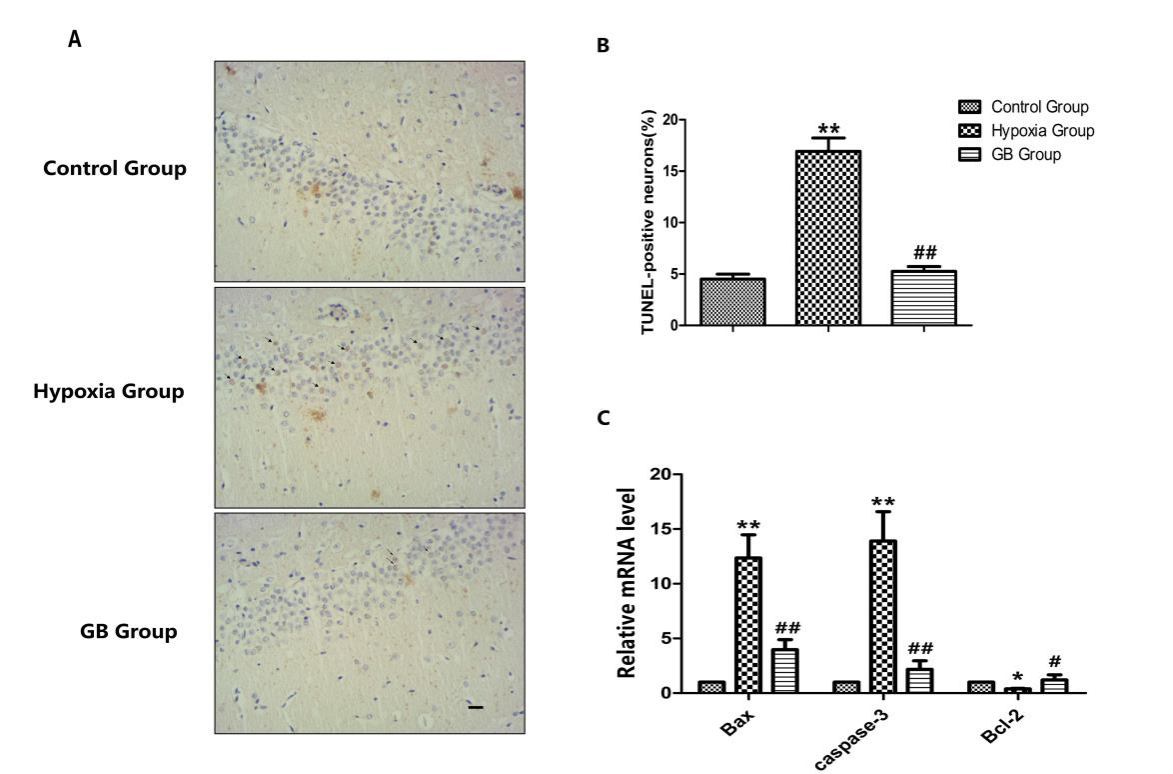
Ginkgolide B (GB) protects neurons against oxidative stress

(A) The activity of superoxide dismutase (SOD). (B) The activity of catalase. (C) The concentration of glutathione (GSH). (D) The concentration of malondialdehyde (MDA). The results are expressed as mean±SD. *P<0.05, **P<0.01 vs. control group; #*P*<0.05, ##*P*<0.01 vs. hypoxia group

***Section preparation***

After six days in the animal decompression chamber, rats were perfused with 0.9% saline followed by 4% paraformaldehyde after being anesthetized. The brains were removed and postfixed overnight in 4% paraformaldehyde. The hippocampi were separated, embedded in paraffin, and sectioned. The sections were dewaxed and rehydrated in xylol (Sigma, Germany) and a graded series of 100%, 90%, 80%, and 70% ethanol for subsequent staining procedures. 

***Hematoxylin and eosin (HE) staining***

The hypothalamus sections were stained with Hematoxylin (Solarbio, G1120) for 10 min, and washed with running water for 5 min. Next, the sections were placed into 0.5% acid alcohol for 10 sec for differentiation (lightening of staining, especially outside the nucleus) and washed with running water for 5 min. The sections were then stained with eosin (Solarbio, G1120) for 20 sec. After washing with running water for 5 min, all stained sections were dehydrated in a graded series of 70%, 80%, 90%, and 100% ethanol and cleared in xylol for 15 min. After mounting, the sections were examined using a light microscope.

***Nissl staining***

Nissl staining was performed using a Nissl Staining Solution kit (Solarbio, cat. G1430). The sections were placed in cresyl violet stain at 56 ^°^C for 1 hr and washed with deionized water. The sections were then placed in Nissl Differentiation Solution for 45 sec. All stained sections were dehydrated in a graded series of 70%, 80%, 90%, and 100% ethanol and cleared in xylol for 15 min. After mounting, the sections were examined using a light microscope. 

***Terminal deoxynucleotidyl transferase-mediated dUTP end labeling (TUNEL)***

TUNEL staining was performed using the Colorimetric TUNEL Apoptosis Assay Kit (Beyotime, cat.C1091). Endogenous peroxidase activity was blocked by incubating with 0.3% H_2_O_2_ in methanol for 30 min. For permeabilization of tissue, the sections were incubated with 20 μg/ml proteinase K in 10 mM Tris HCl (pH 7.5) for 10 min at room temperature. The sections were then incubated in 50 μl TUNEL solution for 1 hr at 37^ °^C. After incubation with converter POD (peroxidase), and HRP (horseradish peroxidase) for 1 hr at 37 ^°^C, the sections were incubated with DAB (3,3–diaminobenzidine, Beyotime, cat.C1611) chromogen at 37 ^°^C. Cells were counterstained with hematoxylin for 20 sec. All stained sections were dehydrated in a graded series of 70%, 80%, 90%, and 100% ethanol and cleared in xylol for 15 min. After mounting, the sections were examined using a light microscope, and the number TUNEL-positive CA1 neurons was counted carefully in five sections per animal. Cell counts from the hippocampus on each of the five sections were averaged to provide the mean value (18). Apoptosis index (AI) was calculated according to the following formula: AI = (number of apoptotic cells/ total cells) × 100%. 

***Quantitative real-time PCR***

Total RNA was extracted from hippocampus homogenate by using an RNA Extraction Kit (TaKaRa, Cat.9767), and cDNA was synthesized using the PrimeScript^® ^1st Strand cDNA Synthesis Kit (TaKaRa, Cat.D6110A). The apoptosis markers were quantified using specific primers: 5ʹ-TATCCAATCCTGTGCT GCTAT-3′ and 5′-CTCTTGCGGAGTATTTGTGC-3′ for Bcl-2,5′-CTG-3′ and 5′-AGGAAAACGCATTATAGACCAC-3′ for Bax, 5′-TGACTGGAAAGCCGAAACTCCGAAACTC-3′ and 5′-AGCCTCCACCGGTATCTTCT-3′ for caspase-3. The thermal cycling conditions were as follows: 1 cycle at 95 ^°^C for 10 min; 30 cycles at 94 ^°^C for 15 sec, 55 ^°^C for 30 sec, and 72 ^°^C for 30 sec; and 1 cycle at 72 ^°^C for 10 min. The PCR reactions were performed with a SYBR green-based system (TaKaRa, Cat.RR82LR), and the gene fold changes were calculated using the (2^−ΔΔCt^) method.

***Oxidative stress index test***

Animals of all experimental groups were sacrificed by cervical dislocation after intraperitoneal injection of 3% pentobarbital sodium (40 mg/kg), and the hippocampus was resected. The hippocampus was homogenized with cold normal saline (NS), centrifuged (4 ^°^C, 12,500 *g*, 10 min), and the supernatant was collected for assays. 

MDA (Beyotime, cat.S0131), SOD (Solarbio, cat. BC0175), catalase (Beyotime, cat.S0051), and GSH (Beyotime, cat.S0053) assays were performed using assay kits, according to manufacturers’ instructions.

***Statistical analysis***

All values were presented as mean±SD. Statistical analysis was performed using one-way analysis of variance (ANOVA) by SPSS software (version 15.0; Chicago, IL, USA). An effect was considered statistically significant if the* P*-value was less than 0.05 (*P*<0.05).

## Results

***The protective effect of GB on hippocampal neuron damage induced by acute hypoxia***

HE staining showed that, in rats of the untreated control group (group I), neurons and neural tissue were intact and had normal morphology (Figure 1). These cells had integrity, regular structure, high cell-density, and were aligned in the CA1, CA2, and dentate gyrus (DG) areas. The pyramidal cells had round nuclei, prominent nucleoli, and clear cytoplasm. However, in rats exposed to 6000 m high altitude (group II), neuronal cells showed morphological changes including irregular cell contour, edema, low cell-density, irregular nuclei with unclear nucleolus and loose chromatin. Hypoxia-induced injury to neuronal cells was ameliorated by prior GB treatment (group III). 

Nissl staining yielded similar results. As shown in Figure 1, after acute exposure to 6000 m simulated altitude, pronounced neuronal loss was observed in the hippocampal CA1 area of rats of group II. This damage could be mitigated by treatment with GB, and visible Nissl bodies were observed in animals of group III.

These results indicate that acute hypoxia exposure induced neuronal damage in the hippocampus. However, prior intragastric administration of GB (12 mg/kg) could protect hippocampal cells from hypoxia-induced damage. 

***Anti-apoptotic effect of GB on hippocampal neuron damage induced by acute hypoxia***

Using TUNEL staining, apoptosis in the brains of rats in the different groups was observed as follows (Figure 2A): the number of apoptotic cells was increased in the high-altitude group; the GB group had fewer apoptotic cells in the CA1 area. When comparing the AI at the same time point among different groups (Figure 2B), the AI of the GB group was significantly lower than that of the high-altitude group (*P*<0.05). 

We also tested the mRNA level of the apoptosis indices including Bax, Bcl-2, and caspase-3 (Figure 2C). Compared to the control group, Bax and caspase-3 expression increased, and Bcl-2 expression decreased (*P*<0.05) in the high-altitude group. There was no significant difference in apoptotic index expression (*P*>0.1) between the control and GB groups. These results indicate that acute hypoxia exposure leads to apoptosis in hippocampal nerve cells, and that GB may have an anti-apoptotic effect by which it could protect hypoxia-induced brain damage.

***GB treatment protects against oxidative stress in hippocampal neurons exposed to acute hypoxia***

As shown in Figures 3A-D, the activity of SOD and catalase and the concentration of GSH decreased significantly, and the concentration of MDA increased significantly in the high-altitude group compared to the control group (*P*<0.05). In addition, SOD and catalase activity, and GSH concentration increased, and MDA concentration decreased in the GB group compared to the high altitude group (*P*<0.05). Thus, GB likely prevents oxidative stress.

## Discussion

In modern times, more people visit plateaus with minimal conditioning, for work or tourism. The development of medicines to protect the brain under hypoxic conditions is therefore of clinical significance. In this study, we established a mammalian model to simulate high-altitude and hypoxia environments to study the neuroprotective effect of GB on the brain. There are 3 major subfields of the hippocampus that include DG, CA1, and CA3. Previous studies have shown that the CA1 region is an important area that correlates with spatial memory impairment (19, 20). Therefore in this study, the CA1 region was chosen as a major region to identify the apoptosis and neuronal survival conditions in this test.

 GB exerts antioxidant effects by scavenging peroxy radicals (21-23) and lowering ROS and MDA levels (15, 24), and can suppress oxidized low-density lipoprotein (LDL)-induced inflammatory protein expression and inhibit nuclear factor-κB (NF-κB) activation in human endothelial cells (25, 26). Our results similarly suggest that augmentation of the antioxidant defense system by GB eventually leads to quenching of free radicals and reduction of ROS and lipid peroxidation. Oxidative stress can cause mitochondrial damage, complete caspase activation, and ultimately apoptosis in neuronal cells (27). Therefore, the neuroprotective effect of GB is likely mediated by its antioxidant function.

The effective suppression of apoptosis and rescue of germ cells by GB extract in testes exposed to doxorubicin (Dox) has been reported; however, the mechanism responsible for this process remains unclear. GB extract suppresses apoptosis induced by beta-amyloid in neuronal cells by influencing mitochondrial membrane potentials, regulating Bcl-2 family members, and repressing caspase-3 activity (28, 29). GB induced the expression of cytochrome P450 3A4 (CYP3A4) and multidrug resistance protein 1 (MDR1) in a pregnane X receptor (PXR)-dependent manner to exert anti-apoptotic and anti-inflammatory effects in endothelial cells (30). Our results showed an anti-apoptotic effect of GB consistent with those described in the aforementioned studies. We found that GB could reduce the expression of Bax and caspase-3, which are unregulated in apoptotic cells. Therefore, the anti-apoptotic property of GB could also play a role in GB-mediated neuroprotection.

This study has some limitations. First, the animal decompression chamber had to be reset to normal altitude conditions because of daily feeding. The animal model therefore failed to simulate the exact conditions of the human body on a plateau. Second, we used only one dose of GB in our experiment, and did not carry out a dose-response study. Third, we did not investigate the influence of GB on the molecular pathways of apoptosis and oxidative stress. We intend to address these limitations in future studies.

## Conclusion

In summary, we successfully established an animal model for plateau hypoxia, which showed marked neuronal cell damage in brain slices. We showed that GB treatment could protect against hippocampal injury. Finally, we showed that GB could protect neuronal cells against acute hypoxia by mechanisms that include anti-oxidative and anti-apoptotic effects. Our results could provide a basis for the development of novel therapeutic strategies to ensure neuroprotection under acute hypoxia at high altitudes.

## Conflicts of Interest

No benefits in any form have been received or will be received from a commercial party related directly or indirectly to subject of this article.
